# Promoting Attitudes Towards Disability in University Settings: A Quasi-Experimental Study

**DOI:** 10.3390/ejihpe15070119

**Published:** 2025-06-25

**Authors:** Luisa Gámez-Calvo, Margarita Gozalo, Miguel A. Hernández-Mocholí, Jesús Muñoz-Jiménez

**Affiliations:** 1 Training Optimization and Sports Performance Research Group (GOERD), Faculty of Sport Science, University of Extremadura, 10005 Caceres, Spain; suliwan@unex.es; 2 Psychology Laboratory, Faculty of Sport Science, University of Extremadura, 10005 Caceres, Spain; mgozalo@unex.es; 3 Physical Activity and Quality of Life Research Group (AFYCAV), Faculty of Sport Science, University of Extremadura, 10005 Caceres, Spain; mhmocholi@unex.es

**Keywords:** inclusion, attitudes toward disability, educative intervention, university students

## Abstract

Inclusive Education emphasizes equal opportunities for all students by reducing or eliminating barriers that hinder participation and learning, encompassing cultural, social, practical, and political dimensions. In this context, the development of positive attitudes towards disability is a key component for the success of inclusive educational practices, particularly in adapted physical education. Following a prior analysis of the impact of academic curricula on attitudes toward disability, the need to implement training programs focused on attitudes toward disability becomes evident. The aim of this study is to analyze how a training program, with a specific focus on attitudes toward disability, affect the attitudes among university students. A quasi-experimental approach with a control group was employed to evaluate the impact of the training. A total of 137 University students from Extremadura, across Physical Activity and Sport Science and Early Childhood Education programs within the same educational department, participated in this study. The Spanish Attitudes Toward People with Disabilities Scale for Professionals was administered to students at the beginning, after the theoretical intervention, and after having a guided contact with people with disability. Results revealed improvements in attitudes in the social relationships and normalized life dimensions, as well as in the total score, particularly among students from Early Childhood Education. No significant changes were observed in the intervention program dimension. Within-group analysis indicated significant longitudinal improvements in normalized life for both experimental groups, Sports Science and Early Childhood Education students, and in the total score for Early Childhood Education students by the end of the intervention. These findings highlight the importance of targeted training interventions in promoting inclusive attitudes, especially when direct contact with individuals with disabilities is incorporated.

## 1. Introduction

Educational inclusion is a fundamental priority within global educational and social frameworks, aiming to provide all students, regardless of their circumstances, abilities, or challenges, with equal access to learning and development opportunities. This objective extends beyond the classroom, playing a crucial role in healthcare settings, where positive perceptions of disability can significantly impact both the quality of care and the effectiveness of inclusive practices (Shields et al., 2024). However, there remains a lack of comprehensive knowledge about disability, and insufficient education on this subject, which contributes to the persistence of stigmatizing attitudes, especially among professionals working directly with individuals with disabilities (Arias González et al., 2016; Scior & Werner, 2015).

In educational and healthcare professions, such as physical rehabilitation or training, fostering attitudes of empathy, inclusion, and understanding is essential, as these perspectives directly influence the future practices of teachers, trainers, and other specialists (Friedman & VanPuymbrouck, 2021). The Inclusive Education model, which promotes equal learning opportunities and addresses the specific needs of students with disabilities, serves as the foundation for these efforts (Pradhan & Naik, 2024; Erkilic & Durak, 2013). Implementing inclusive education requires actively eliminating barriers to participation by improving accessibility, tailoring instructional materials, and training educators in disability and inclusive practices (Booth & Ainscow, 2018). As the significance of inclusive education continues to grow, so does the challenge of assessing its impact. Evaluating inclusion involves more than just tracking enrollment and academic success rates, it also requires qualitative research through interviews and surveys, despite the subjective nature of such approaches (Krämer et al., 2021; Young & Clerke, 2024)

Developing positive attitudes toward disability is particularly important among university students pursuing degrees in disciplines such as Physical Activity and Sport Science (PASS) and Early Childhood Education (ECE). These perspectives play a key role in fostering inclusive environments and reducing social stigma, with PASS emphasizing in physical activity rehabilitation and training inclusion and ECE focusing on supporting educative inclusion (Babik & Gardner, 2021; Tejero et al., 2022; Hernández-Beltrán et al., 2023). Previous research indicates that students in fields such as education, social sciences, and healthcare generally exhibit more favorable attitudes toward disability than those in technical disciplines like architecture and engineering (Garabal-Barbeira et al., 2018; Polo et al., 2011). However, barriers to inclusion persist, particularly due to rigid curricula and a lack of training in inclusive methodologies (Jardinez & Natividad, 2024).

Studies conducted in Spain suggest that university curricula in programs like Occupational Therapy and ECE contribute to improved attitudes toward disability; however, this positive modification is less evident among students enrolled in PASS programs (Gámez-Calvo et al., 2025). The results of this previous study revealed that while academic curricula may influence attitudes toward disability, university training alone is insufficient to generate meaningful change in these attitudes, underscoring the need to implement specific training programs focused on attitudes toward disability. Prior research suggests that these changes in attitude may result from a combination of academic exposure and personal development over time (García-Fernández et al., 2017; Polo Sánchez et al., 2018). These findings underscore the need of implementing educational strategies tailored to enhance attitudes toward disability at various stages of university education, ensuring that students receive adequate training on this subject during their academic journey. Educative methodologies play a pivotal role in shaping attitudes towards disability, as evidenced by recent research (Gámez-Calvo et al., 2024a). Studies reveal that interventions combining theoretical instruction with practical experiential learning, including both direct and indirect contact with people with disabilities, are particularly effective in promoting positive attitudinal change (Felipe Rello et al., 2018; Reina et al., 2021). Moreover, approaches informed by modern pedagogical frameworks, such as the Theory of Planned Behavior, further enhance this process by fostering empathy and reducing prejudicial views through structured cooperative activities and interactive sessions (Bebetsos et al., 2017). These outcomes highlight that a multifaceted strategy integrating both knowledge and experiential components is essential to effectively improve attitudes towards disability in inclusive educational settings.

As stated above, the measurement of inclusion remains a subject of debate, as some scholars question whether its cultural, social, and psychological dimensions can be fully captured through traditional metrics or if it is better understood as an ideal that resists quantification (Garabal-Barbeira et al., 2018; Tai et al., 2023). A comprehensive assessment requires combining quantitative indicators—such as enrollment, attendance, and academic progression—with qualitative insights drawn from personal experiences collected through interviews and focus groups (Delve & Limpaecher, 2024; Mezzanotte & Calvel, 2023). Instruments such as the “Index for Inclusion” (Mezzanotte & Calvel, 2023) enable educational institutions to evaluate and refine their inclusive practices by examining cultural, policy, and pedagogical dimensions (Ortega et al., 2014; Sandoval, 2002). Furthermore, in the assessment of attitudes toward disability, the use of valid and reliable instruments is crucial for ensuring the rigor of research findings. One of the most widely recognized tools in this field is The Attitudes Towards People with Disabilities Scale (Arias González et al., 2016), which has become a reference point in both national and international studies.

The aim of this study is to provide reliable data on the effectiveness of a training programs in improving university student attitudes toward disability. This training program was integrated into university curricula, particularly within PASS and ECE degrees. To evaluate their effectiveness, a quantitative analysis will be conducted to measure changes in attitudes toward disability, supplemented by an observational and descriptive assessment of the training initiatives. Furthermore, the specific objectives of this study are as follows: (1) to describe and quantify the changes in attitudes towards disability after participating in a specific training program; (2) to analyze the differences in the change in attitudes towards disability based on the training programs; and (3) to identify and quantify the factors that influence the improvement of attitudes towards disability.

## 2. Materials and Methods

### 2.1. Study Design and Ethical Considerations

This study employs a non-equivalent groups quasi-experimental design with an intervention comparison framework, a robust approach frequently utilized in research contexts where random assignment is not feasible. The design involves comparing two groups enrolled on a similar intervention that consists of a general theoretical educative training on disability and attitudes toward disabilities combined with a practical session that includes direct contact with individuals with disabilities. The interventions have been integrated into the university curriculum for PASS and ECE programs. Due to differences in the curriculum structures, the final interventions vary in format and delivery; however, the core content addressing knowledge about people with disabilities and attitudes toward disability remains consistent across both programs. Furthermore, the design involves a control group of first-year students that does not attend any type of specific training about disability.

All data will be collected anonymously. Prior to the start of the training programs, participants were informed about the details of the research, including its potential risks and benefits, and were provided with an informed consent.

The study was conducted according to the guidelines of the Declaration of Helsinki (Gray et al., 1978) and approved by the Ethics Committee of the University of Extremadura (registration code: 151/2022).

### 2.2. Participants

A total of 137 University Students from Extremadura, across PASS and ECE degree programs, participated in this quasi-experimental study. The University of Extremadura is a public institution located in the autonomous community of Extremadura, in Spain. The relatively low cost of living, combined with smaller class sizes and a more personalized educational experience, can positively influence student satisfaction. However, students may also face challenges related to limited international exposure or fewer professional opportunities in the region. The eligibility criteria included all aged and gender participants who were involved in university programs related to PASS and ECE at the University of Extremadura, who provided signed informed consent to the research team.

Participants included final-year students from each program, allowing for a comparative analysis of attitudes towards disability after the training program, and first year students as control group. The sample (*n* = 137) mean age was 21.6 years (SD = 3.78), the sociodemographic characteristics and the characteristics related to contact with people with disabilities are shown in Table 1.

### 2.3. Sample Procedure

The sample was gathered using a cluster sampling approach, selecting naturally formed groups of students who consistently attended classes in the chosen university programs. The data collection process was structured around four essential documents: the informed consent form, the measurement scale, the list of participating students, and the interviewers’ guidelines. The instructor assisting with data collection was contacted via email, where the study’s objectives were outlined. The data collection was conducted by experienced researchers who received specific training for this study. Students provided informed consent before completing the questionnaire in person via Google Forms during class hours, allowing them to seek clarification on any items to ensure full comprehension.

### 2.4. Measures and Instruments

To analyze university students’ attitudes toward disability, several key variables were considered:

- Sociodemographic Variables (gender, age, higher level of prior education, and degree program), variables related to contact with people with disabilities (prior contact with people with disability, reason for contact, frequency of interaction, type of disability, and emotional response in presence of people with disabilities), and variables related to knowledge about disabilities (physical disability, sensory disability and intellectual disability)
- Assessment of Attitudes Towards Disability: Score on the Attitudes Towards Disability Scale (Arias González et al., 2016).

To evaluate university students’ attitudes toward disability, the study employed the Attitudes Towards People with Disabilities Scale (Arias González et al., 2016). This instrument was originally validated with a large sample of 976 professionals—primarily from the education and healthcare fields—who were selected due to their frequent interaction with individuals with disabilities. The scale is recognized for its robust psychometric properties, making it a reliable tool for research purposes. It includes 31 items, distributed across three key dimensions. The first factor (SR), “*Social and Interpersonal Relationships with People with Disabilities*” (13 items), measures emotional, cognitive, and behavioral components in personal and social encounters. The second factor (NL), “*Normalized Life*” (13 items), addresses the belief in the right of people with disabilities to live independently and equitably, with access to the same opportunities as others. The third factor (IP), “*Intervention Programs*” (5 items), gauges support for inclusive initiatives, focusing on both their effectiveness and economic viability. This scale is among the most widely accepted instruments for measuring attitudes toward disability, owing to its high reliability and scientific rigor (Simón Medina et al., 2024). Table 2 presents a comparison of Cronbach’s alpha values between the original instrument and the current dataset. A Cronbach’s alpha above 0.7 indicates satisfactory reliability, confirming that the data gathered in this study reliably reflect participants’ attitudes (Taber, 2018). However, the alpha coefficient for Factor 3 fell below this threshold, suggesting reduced internal consistency in responses related to support for intervention programs and their perceived financial feasibility. Analyzing Cronbach’s alpha at different measurement moments is essential to ensure the internal consistency and reliability of the questionnaire over time. By comparing Cronbach’s alpha values obtained at each measurement point with those reported in the original questionnaire, researchers can assess the stability and robustness of the scale across different conditions and samples (Bonett & Wright, 2015).

Based on Appendix B “Norms” of the original questionnaire, cutoff points were established to interpret direct scores on the three factors of the scale in terms of “unfavorable”, “medium”, and “positive” attitudes toward disability. For Factor 1 “*Social and Interpersonal Relationships*”, scores below 41 indicate less favorable attitudes, scores from 42 to 49 reflect medium attitudes, and scores above 50 suggest positive attitudes. In Factor 2 “*Normalized Life*”, scores under 41 are interpreted as less favorable, 42 to 47 as medium, and above 48 as positive. For Factor 3 “*Intervention Programs*”, scores below 15 reflect less favorable views, 16 to 18 are medium, and above 19 indicate positive attitudes. Overall scale scores below 102 suggest less favorable attitudes, scores between 103 and 115 are considered average, and scores above 116 are interpreted as positive attitudes toward people with disabilities.

### 2.5. Statistical Analysis

Data analysis was performed using the open statistical software R (v4.3.3) (Fox & Weisberg, 2019; R Core Team, 2023). Initially, an exploratory and descriptive analysis of the sample was carried out, to ensure data quality and characterize the distribution of sociodemographic variables and stablish an initial profile of the participants. To assess variability at different levels and determine the impact of specific variables (academic year, gender, and contact with people with disabilities) on attitudes toward disability, a linear mixed model was implemented. This statistical approach allowed for the inclusion of both, fixed and random effects, thus facilitating the analysis of how these variables influenced the observed changes (Pinheiro & Bates, 2000).

Normality of the data was assessed using the Shapiro–Wilk test (Shapiro & Wilk, 1965). Given that the assumption of normality was not met, non-parametric techniques were used to compare outcomes between groups. The Kruskal–Wallis’s test was applied to detect overall differences between groups (Kruskal & Wallis, 1952). Subsequently, Dunn’s post hoc test with Bonferroni adjustment was performed to identify specific comparisons showing significant differences. The same procedure was used to analyze the effects of the intervention within the same group (Jean Dunn, 1964).

### 2.6. Description of the Intervention

The intervention program was structured based on four dimensions: Attitudes, Resources (virtual, methodological, material resources, and practical experiences), Methodologies, and Evaluation, with the aim of providing participants with skills about the inclusion of people with disabilities in different areas related to education and sports training. The program comprises 20 h of theoretical and practical training, followed by two practical experiences involving direct interaction with individuals with disabilities, lasting 2 h each. This training provided general knowledge on disability and specific interventions aimed at addressing attitudes toward disability. For more details see Appendix A.1. Furthermore, this study includes the educational and curricular plans related to disability of PASS and ECE, which aimed to assess the potential influence of academic training programs on attitudes, all the competences mentioning “disability”, “diversity”, or “inclusion” were included, for more details see Appendix A.2.

The theoretical and practical training for students in the ECE and PASS programs was conducted during the 2022–2023 academic year as part of the ECE and PASS undergraduate degrees. On one hand, the ECE program took part at the Faculty of Education and Psychology, University of Extremadura, Badajoz Campus. The training was integrated into the course titled “The Body and Motor Skills in Detecting and Addressing Individual Differences” (Course Code: 501606), an optional fourth-year subject. The theoretical intervention consists of six sessions, each lasting approximately 120 min, along with four practical sessions of the same duration, all aligned with the official schedule of the degree program. On the other hand, the PASS was implemented at the Faculty of Sport Sciences, University of Extremadura, Cáceres Campus. The training was included within the mandatory fourth-year course “Adapted Physical Activity and Sport” (Course Code: 500297). The theoretical intervention consists of thirteen sessions, each lasting approximately 60 min, along with six practical sessions of 90 min, all aligned with the official schedule of the degree program.

The planning of contents, resources, and the activities conducted in the intervention could be consulted in Annex 1. However, due to the complexity and extensive content of the sessions, various modifications were made during their implementation to better align with the academic program of each degree. Participants were provided with all materials and supplementary explanations via the Virtual Campus of the University of Extremadura, the official platform for academic activities, accessible to all instructors and participants involved in this training program.

Furthermore, the timing of the intervention was the same for both experimental groups, lasting one month. It began in the first semester of the academic year, starting in the third week of September and ending in October. The pre-test was administered to all groups on the day the intervention began. The second measurement was conducted only with the experimental groups, immediately after the conclusion of the theoretical and practical intervention. Finally, the third evaluation took place following the direct contact with individuals with disabilities in December in the experimental groups, coinciding with the final data collection for the control group, which did not receive any type of intervention.

## 3. Results

The statistical analysis revealed notable findings regarding the effects of the educational intervention on attitudes toward disability, although these effects were not significant across all variables. First, a descriptive analysis of the sample was conducted (Table 3), including the mean scores and standard deviations for each factor of the questionnaire, categorized by academic degree. Additionally, each result was assigned a label indicating a favorable, neutral, or unfavorable attitude, based on the obtained scores compared to those from the original version of the questionnaire.

A linear mixed-effects model was subsequently used to evaluate the impact of variables such as academic year, gender, and contact with individuals with disabilities on attitudes. This model allows for the inclusion of both fixed effects (experimental groups) and random effects (individual variability and variability across repeated measures), providing an estimate of the influence of independent variables while accounting for the internal structure of the data and controlling for intra-individual variability. After analyzing different specifications of the linear mixed model, it was decided to include only academic degree as an influential variable, as gender and contact with disability did not explain the observed changes across measurement points.

**Figure 1:**
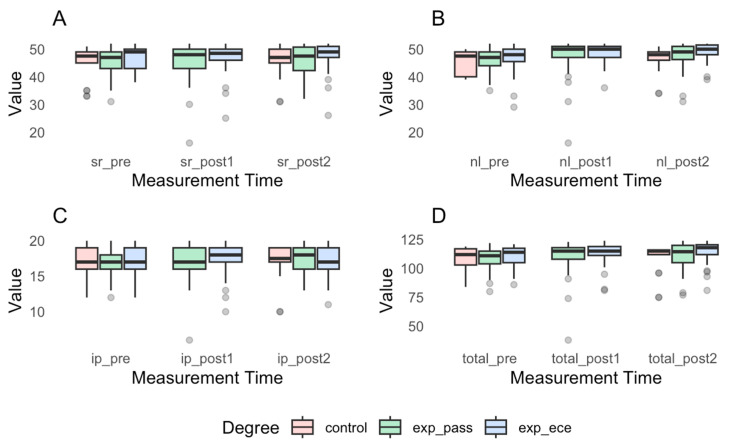
Scores by measurement time and experimental group for each factor and total score of the questionnaire. (**A**) refers to the Social and Interpersonal Relationships with People with Disabilities factor; (**B**) to the Normalized Life factor; (**C**) to the Intervention Programs factor; and (**D**) to the Total Score.

Figure 1 presents the graphs of the scores for each factor and the total score of the questionnaire based on the time of measurement and the experimental group. Graph A corresponds to the Social and Interpersonal Relationships factor, Graph B to the Normalized Life factor, Graph C to the Intervention Programs factor, and Graph D to the Total Score.

Since the residuals of the mixed-effects model did not meet the assumption of normality (Shapiro–Wilk, *p* < 0.001), but did show homogeneity of variances (Levene, *p* > 0.05), non-parametric tests (Kruskal–Wallis and Dunn’s post hoc test with Bonferroni correction) were used for group comparisons over time. Cliff’s Delta was employed to calculate the effect size, providing a non-parametric measure of the magnitude of differences between groups. According to the established thresholds proposed by Cliff (1993), values below 0.14 indicate a negligible effect, between 0.14 and 0.33 a small effect, between 0.33 and 0.47 a moderate effect, and above 0.47 a large effect.

### 3.1. Group Comparison

The comparison between groups for each factor of the questionnaire, as well as for the total score, is presented in Table 4. This table includes the standard deviation, the *p*-value obtained from the Kruskal–Wallis test, the adjusted *p*-value from Dunn’s post hoc comparisons (with Bonferroni correction), and an indication of statistical significance. These results allow for a detailed analysis of how the different groups varied in their attitudes toward disability across the measured dimensions.

#### 3.1.1. Factor 1: Social and Interpersonal Relationships with People with Disabilities

No significant differences were observed in the initial scores, indicating that all groups started at a similar level of attitudes toward disability. Regarding the Cliff’s Delta, all comparisons yielded negligible or small effects. However, significant differences emerged in the second measurement (Post 2) between the experimental groups: the ECE group scored significantly higher than the PASS group (*p* = 0.028; δ = −0.176, small effect). No significant differences were found between the control group and the experimental groups in any of the measurements.

#### 3.1.2. Factor 2: Normalized Life

No significant differences were observed in the initial scores, indicating that all groups started at a similar level of attitudes toward disability. Regarding the Cliff’s Delta, moderate effects were observed in comparisons between Control and ECE both at Pre (δ = −0.364) and Post 2 (δ = −0.364) measurements; although, only in the second measurement (Post 2), significant differences were found between the control group and the ECE experimental group (*p* = 0.008), suggesting a more positive attitude in the latter group toward the normalization of life for individuals with disabilities. However, no significant differences were found between the two experimental groups or between the control group and the PASS experimental group.

#### 3.1.3. Factor 3: Intervention Programs

No statistically significant differences were found between the groups in any of the three measurement points for this factor, with all groups showing average attitudes in their scores. Regarding the Cliff’s Delta, Factor 3, presented consistently negligible effects across all time points. This suggests that the intervention did not effectively improve attitudes toward supporting initiatives that promote inclusion, integration, and the financial feasibility of intervention programs or treatments for people with disabilities. It is worth noting that this factor includes the fewest items and exhibits the lowest internal consistency.

#### 3.1.4. Total Score

Significant differences were observed in the total score at the second measurement between the control group and the ECE experimental group (*p* = 0.027; δ = −0.277, small effect), indicating a greater impact of the intervention on the latter group. In addition, the Cliff’s Delta shows small effect sizes in most group comparisons.

### 3.2. Intra-Group Comparison over Time

The same non-parametric techniques were subsequently used to analyze intra-group differences over time. The intra-groups comparison for each factor of the questionnaire, as well as for the total score, is presented in Table 5. This table includes the standard deviation, the *p*-value obtained from the Kruskal–Wallis test, the adjusted *p*-value from Dunn’s post hoc comparisons (with Bonferroni correction), and an indication of statistical significance. These results allow for a detailed analysis of how each group varied in their attitudes toward disability over the time.

#### 3.2.1. Factor 1: Social and Interpersonal Relationships with People with Disabilities

No statistically significant changes were observed over time in any of the groups, also, regarding the Cliff’s Delta, all comparison showed negligible effects.

#### 3.2.2. Factor 2: Normalized Life

The PASS experimental group showed significant improvements between the first and second measurements (*p* < 0.001; δ = 0.368, medium), as well as between the first and third (*p* = 0.001, δ = 0.325, small). Similarly, the ECE group demonstrated a significant improvement between the first and third measurements (*p* = 0.004; δ = 0.325, small effect), indicating a positive evolution in attitudes toward the normalization of life for people with disabilities. Moreover, regarding the Cliff’s Delta, small to medium effects were observed in the rest of the comparisons.

#### 3.2.3. Factor 3: Intervention Programs

No significant within-group differences were found over time for this factor; furthermore, regarding the Cliff’s Delta, all comparison showed negligible effects.

#### 3.2.4. Total Score

Only the ECE group showed a significant improvement in the total score between the first and third measurements (*p* = 0.015; δ = 0.242, small effect), suggesting a favorable change in overall attitudes following the intervention. In addition, regarding the Cliff’s Delta, small effects were observed in the all the comparisons.

## 4. Discussion

This study aimed to provide reliable data on the effectiveness of a training programs in improving university student attitudes toward disability, specifically targeting those enrolled in ECE and PASS degrees. Consistent with previous research (Atoche-Silva et al., 2021; Estany & Bravo, 2017), university students generally display medium to positive attitudes toward individuals with disabilities. However, there are still areas requiring improvement. Prior studies have highlighted gaps between the academic training provided and the professional competencies required to support individuals with disabilities effectively (Gámez-Calvo et al., 2025; Hernández-Beltrán et al., 2023). This highlights the need to strengthen University education around attitudes toward disability; therefore, this intervention has been proposed with the aim of improving university students’ attitudes toward disability.

Various review studies have analyzed the methodologies used to design and implement training programs focused on attitudes toward disability and educational inclusion. These studies emphasize that the most effective strategies in such programs include contact with individuals with disabilities and experiential learning (Gámez-Calvo et al., 2024a; Reina et al., 2021; Young & Clerke, 2024). Accordingly, the training program proposed in this study is grounded in practical and experiential learning. Participants not only learn about various types of disabilities and effective ways to support individuals with them, but also actively engage in hands-on experiences. These include role-playing activities that simulate the perspectives of people with disabilities, as well as structured, guided interactions with individuals with disabilities within the academic setting. Through shared activities, participants develop a deeper understanding and empathy, fostering more inclusive attitudes and behaviors.

This intervention program was integrated into university curricula and aims to compare its effectiveness in two different groups. The researchers hypothesize that the theoretical and practical components play a significant role in changing attitudes, expecting a substantial improvement in attitudes among students receiving this specific training. This aligns with previous research highlighting the importance of education about attitudes toward disability to promote more inclusive educational environments (López & Moreno, 2019; Reina et al., 2016; Shields et al., 2024). Additionally, studies have shown that educational interventions emphasizing direct interaction and understanding of disability are effective in fostering better attitudes among students (Garabal-Barbeira et al., 2018; Polo Sánchez et al., 2018). It has also been noted that direct contact with individuals with disabilities is a key factor in fostering positive changes in participants’ attitudes toward disability (Polo et al., 2011; Reina et al., 2022). The impact of this contact may be mediated by the quality of the interaction and the context in which it takes place, suggesting the need to carefully design these activities. Furthermore, the intervention proposed in this article is sustained in previous scientific evidence that supports the use of experiential learning, reflective practices, and structured contact with individuals with disabilities as effective strategies for attitude change.

Similarly to previous studies (Felipe Rello et al., 2018; López & Moreno, 2019; Martínez Martín & Bilbao León, 2011), this work used a survey to analyze university students’ attitudes toward disability. Attitudes were measured using the Attitudes Toward People with Disabilities Scale (Arias González et al., 2016). Additionally, prior studies (Gámez-Calvo et al., 2025) have examined whether university degree programs are associated with differences in students’ attitudes toward disability. These studies revealed significant differences in baseline scores between students in ECE and PASS, with ECE students exhibiting significantly more positive attitudes. However, in the study sample the base scores did not show significant differences, so it can be stated that the groups start from a similar initial level of attitudes.

The results of the comparative analysis between groups reveal an uneven impact of the educational intervention depending on the university degree and the factor of the attitudes toward disability questionnaire evaluated. Regarding the first factor “*social and interpersonal relationships with people with disabilities*”, significant differences were only detected in the second measurement between the experimental groups, with the ECE group showing more favorable attitudes compared to the PASS group. However, no significant differences were observed between the experimental groups and the control group, suggesting that the intervention may have had a limited effect or one conditioned by uncontrolled factors, such as students’ prior disposition toward the topic or the specific educational context. These findings align with previous studies that report a greater attitude toward inclusion among students in education-related degrees, such as ECE (Garabal-Barbeira et al., 2018; López & Moreno, 2019), and reinforces the notion that the impact of training programs may vary according to students’ professional profiles (Polo et al., 2011). In relation to the second factor “*Normalized life*”, significant differences were identified in the second measurement between the control group and the ECE experimental group, suggesting an improvement in attitudes toward the full inclusion of people with disabilities in everyday contexts. However, this improvement was not replicated in the PASS group, which may reflect differences in how the intervention was experienced across degree programs or in the relevance of the content to their personal future professional practice.

It is worth noting that, despite the limited intergroup differences, the intra-group analysis revealed a significant positive evolution in attitudes toward normalized life in both experimental groups over time, suggesting that such changes may require a progressive process of reflection and maturation, beyond the immediacy of the intervention. Conversely, the third factor “*Intervention programs*” did not show significant improvements either between groups or over time. Furthermore, topics such as funding or the feasibility of intervention programs may seem more abstract or distant to students in PASS or ECE programs, limiting their ability to develop strong attitudes on the matter, as in prior research this factor has a greater punctuation in health-related students (Friedman & VanPuymbrouck, 2021; Gámez-Calvo et al., 2025; VanPuymbrouck & Friedman, 2020). Previous research has already emphasized the need to contextualize this type of content through experiential activities or real-life case studies to foster greater student engagement (Reina et al., 2021; Young & Clerke, 2024). As for the overall questionnaire score, only the ECE group showed significant improvements in the second measurement compared to the control group and in the intra-group comparison over time, suggesting that the intervention had a more effective global impact in this group. These results support the study’s initial hypothesis that specific training on attitudes toward disability could lead to more evident positive changes in students whose future professional roles are directly linked to inclusive settings or are related to attending people with disabilities. Nevertheless, the modest effects observed call for reflection on the need to strengthen the design of such interventions by ensuring longer durations, longitudinal follow-up, and contextualization based on students’ academic and professional profiles. In this regard, the transversal and sustained integration of disability-related content into university curricula emerges as a key strategy for fostering long-term inclusive attitudes (Felipe Rello et al., 2018; Moreno Pilo et al., 2022). Furthermore, the differences observed between ECE and PASS students prompt consideration of the various factors that influence attitudes toward disability and perceptions of individuals with disabilities. In line with previous research (Babik & Gardner, 2021), some of these factors include the type of disability, with more favorable attitudes typically directed toward individuals with solely physical disabilities, and the age of the person with a disability, as attitudes tend to be more positive toward children than adults. This may be attributed to perceptions of innocence or vulnerability commonly associated with childhood. These factors may help explain why ECE students demonstrate more favorable attitudes, as both their current experiences and future professional trajectories are likely to involve working more frequently with young children in early childhood education settings.

The study presents several strengths, such as an evidence-based design that integrates both theoretical and practical components and employs validated measurement tools like the Attitudes Toward People with Disabilities Scale (Arias González et al., 2016). Furthermore, few previous interventions have explored this area, and there is often an implicit assumption that the university student population is homogeneous, with changes resulting from such programs occurring uniformly, regardless of students’ degree programs or other contextual factors. In contrast, we considered it essential to examine whether pursuing different academic degrees influences how students respond to this type of intervention. The program’s comparative approach, contrasting a specific theoretical module on disability and attitudes toward disability between two different groups, provides valuable insights into the role of targeted education in fostering positive change, as mentioned in prior research (Gámez-Calvo et al., 2024b; Reina et al., 2021; Young & Clerke, 2024). However, weaknesses such as reliance on self-reported data, with possible uncontrolled external factors, and a limited sample scope may affect the generalizability of the findings. While the quasi-experimental design is appropriate for the study’s objectives, the lack of random assignment limits the ability to draw definitive causal conclusions. Without randomization, there is a greater risk of selection bias and the influence of confusing variables. However, due to ethical considerations and the nature of the study, implementing random assignment was not feasible. On the other hand, regarding the sample size, it could represent a limitation that may affect the generalizability of the findings, as it may not fully capture the diversity of experiences or outcomes in broader populations. Another potential limitation is the relatively low Cronbach’s alpha for Factor 3 (“Intervention Programs”), which, as in the original validated questionnaire, falls below the accepted threshold of 0.7. This indicates lower internal consistency in responses related to this factor. However, the results obtained in this study are consistent with those of the original validation, supporting the comparability and relevance of the findings. Furthermore, Cliff’s Delta was employed to assess the magnitude of differences between groups and over time, showing negligible or small effects, indicating modest practical differences despite some statistically significant findings. These results suggest that while the interventions produced statistically detectable changes, the overall magnitude of these changes was limited, which may limit the practical implication of the findings. Opportunities for improvement include the integration of comprehensive content on disability and inclusive education into university curriculum, the potential for long-term impact studies, and the enhancement of experiential learning opportunities through well-designed direct contact activities (Garabal-Barbeira et al., 2018; Polo et al., 2011; Polo Sánchez et al., 2018; Shields et al., 2024). Despite these advantages, threats remain in the form of variable quality in direct interactions with individuals with disabilities, possible institutional resistance to curriculum changes, and concerns regarding external validity due to the study’s specific regional and disciplinary focus. For future research, the long-term impact of training programs on attitudes toward disability should be explored to determine whether the knowledge acquired is effectively applied in participants’ professional performance. Future research with larger and more diverse samples would strengthen the applicability of these results. Moreover, specific programs on attitudes toward disability should be designed for different professional fields to ensure society is increasingly prepared to address and understand diversity, reducing and eliminating the various barriers faced by individuals with disabilities.

## 5. Conclusions

The results of this study indicate that the educational intervention had a notable impact on certain dimensions of attitudes toward disability. Although significant improvements were not observed across all evaluated factors, specific progress was noted, particularly within the ECE experimental group, which showed a positive evolution in the “Normalized Life” dimension as well as in the total questionnaire score. These findings suggest that the training received may have contributed to greater awareness and attitude change in this group. However, the lack of significant effects in the factor related to support for intervention programs highlights the need to design more targeted and extended interventions to foster broader and more sustained changes.

The document emphasizes the need to design training programs that integrate both approaches in a complementary approach, as explicit education prepares students to interpret and value experiential and contact-based experiences more deeply. In practical terms, these findings can guide educational institutions in implementing more effective policies and training programs, contributing to the development of professionals’ attitudes toward disability to address the challenges of an inclusive society. Furthermore, future research should focus on evaluating the sustainability of these improvements over time and exploring how these attitudes influence professional practices once students enter the labor market.

## Figures and Tables

**Table 1:** Sociodemographic characteristics of the sample (*n* = 137).

Variable	Categories	*n*	%
Intervention program	Control	20	14.6%
	Physical Activity and Sport Sciences	66	48.2%
	Early Childhood Education	51	37.2%
Gender	Female	85	62.0%
	Male	52	38.0%
Contact with Disability	Yes	44	32.0%
	No	93	67.9%
Contact Reason *	Familiar	8	18.2%
	Laboral	16	36.4%
	Care Intervention	2	4.5%
	Leisure and Friendship	0	0.0%
	Other	18	40.9%
	Various	3	6.8%
Contact Frequency *	Every Day	3	6.8%
	Several Times a Week	14	31.8%
	Several Times a Month	21	47.7%
	Less than once a Mont	10	22.7%
Type of Disability *	Physical Disability	17	38.6%
	Mental Illness	12	27.3%
	Sensory Disability	10	22.3%
	Other types	5	11.4%
Feeling in presence of people with disability	Very Comfortable	33	24.1%
	Quite Comfortable	62	45.3%
	Indifferent	39	28.5%
	Quite Uncomfortable	2	1.5%
	Very Uncomfortable	1	0.7%

Notes: The variables marked with * correspond to a sample of 44 individuals who reported having had prior contact with people with disabilities. It is important to note that each participant could select more than one option, as they may have experienced different types of contact. Therefore, the percentages for those variables are calculated based on the total of these 44 participants.

**Table 2:** Cronbach’s alpha coefficient.

		Data Collected		
Factor	Original Questionnaire	Pre	Post 1	Post 2
SR	0.858	0.816	0.868	0.872
NL	0.822	0.798	0.908	0.840
IP	0.603	0.497	0.652	0.589
Total Score	0.928	0.889	0.936	0.921

**Table 3:** Descriptive analysis of the sample.

	Factor 1: “Social and Interpersonal Relationships with People with Disabilities”								
	Pre-test			Post-test 1			Post-test 2		
Degree	Mean	Sd	Attitudes	Mean	Sd	Attitudes	Mean	Sd	Attitudes
Control	45.400	6.09	Medium	N/A	N/A	N/A	45.60	6.14	Medium
PASS	45.818	4.70	Medium	45.79	6.41	Medium	45.95	5.36	Medium
ECE	46.70	4.32	Medium	46.97	5.14	Medium	48.23	4.76	Medium
	Factor 2: “Normalized Life”								
	Pre-test			Post-test 1			Post-test 2		
Degree	Mean	Sd	Attitudes	Mean	Sd	Attitudes	Mean	Sd	Attitudes
Control	45.60	4.45	Medium	N/A	N/A	N/A	46.40	4.86	Medium
PASS	46.13	3.72	Medium	47.81	6.10	Medium	48.00	4.38	Favorable
ECE	46.92	4.74	Medium	48.93	3.22	Favorable	49.31	3.06	Favorable
	Factor 3: “Intervention Programs”								
	Pre-test			Post-test 1			Post-test 2		
Degree	Mean	Sd	Attitudes	Mean	Sd	Attitudes	Mean	Sd	Attitudes
Control	17.00	2.38	Medium	N/A	N/A	N/A	16.90	2.65	Medium
PASS	17.07	1.80	Medium	17.03	2.50	Medium	17.26	2.15	Medium
ECE	17.00	1.91	Medium	17.37	2.08	Medium	17.31	2.03	Medium
	Total Punctuation								
	Pre-test			Post-test 1			Post-test 2		
Degree	Mean	Sd	Attitudes	Mean	Sd	Attitudes	Mean	Sd	Attitudes
Control	108.00	11.58	Medium	N/A	N/A	N/A	108.90	12.99	Medium
PASS	109.03	8.75	Medium	110.64	14.03	Medium	111.21	10.68	Medium
ECE	110.62	9.59	Medium	113.28	8.92	Medium	114.86	8.38	Medium

Notes: N/A: Not applicable.

**Table 4:** Comparison of groups on each factor and total score of the disability attitudes questionnaire.

Factor 1: “Social and Interpersonal Relationships with People with Disabilities”							
Measurement moment	Group Comparison	Sd	*p*-value	P_adj	Sig	Cliff’s Delta	Mg
PRE	Control vs. PASS	−0.1538	0.8777	1.000	Ns	−0.015	Negligible
	Control vs. ECE	0.7338	0.4630	1.000	Ns	−0.198	Small
	PASS vs. ECE	1.249	0.2116	0.634	Ns	−0.176	Small
POST1	PASS vs. ECE	0.8843	0.3765	0.376	Ns	−0.176	Small
POST2	Control vs. PASS	0.2358	0.8135	1.000	Ns	−0.015	Negligible
	Control vs. ECE	2.0634	0.0390	0.117	Ns	−0.198	Small
	PASS vs. ECE	2.5972	0.0093	0.028	*	−0.176	Small
Factor 2: “Normalized Life”							
Measurement moment	Group Comparison	Sd	*p*-value	P_adj	Sig	Cliff’s Delta	Mg
PRE	Control vs. PASS	0.1313	0.8954	1.000	Ns	−0.200	Small
	Control vs. ECE	1.4210	0.1553	0.465	Ns	−0.364	Medium
	PASS vs. ECE	1.8310	0.0670	0.201	Ns	−0.146	Negligible
POST1	PASS vs. ECE	0.3334	0.7387	0.738	Ns	−0.147	Negligible
POST2	Control vs. PASS	1.9380	0.0526	0.157	Ns	−0.200	Small
	Control vs. ECE	3.0008	0.0026	0.008	**	−0.364	Medium
	PASS vs. ECE	1.5932	0.1111	0.333	Ns	−0.147	Negligible
Factor 3: “Intervention Programs”							
Measurement moment	Group Comparison	Sd	*p*-value	P_adj	Sig	Cliff’s Delta	Mg
PRE	Control vs. PASS	−0.0238	0.9810	1.000	Ns	−0.006	Negligible
	Control vs. ECE	−0.1523	0.8789	1.000	Ns	−0.026	Negligible
	PASS vs. ECE	−0.1829	0.8548	1.000	Ns	−0.022	Negligible
POST1	PASS vs. ECE	0.64506	0.5188	0.5188	Ns	−0.022	Negligible
POST2	Control vs. PASS	0.24622	0.8055	1.000	Ns	−0.006	Negligible
	Control vs. ECE	0.27649	0.7821	1.000	Ns	−0.026	Negligible
	PASS vs. ECE	0.05417	0.9567	1.000	Ns	−0.022	Negligible
Total Punctuation							
Measurement moment	Group Comparison	Sd	*p*-value	P_adj	Sig	Cliff’s Delta	Mg
PRE	Control vs. PASS	−0.1410	0.887	1.000	Ns	−0.097	Negligible
	Control vs. ECE	0.9602	0.336	1.000	Ns	−0.277	Small
	PASS vs. ECE	1.5519	0.120	0.362	Ns	−0.154	Small
POST1	PASS vs. ECE	0.6009	0.547	0.547	Ns	−0.154	Small
POST2	Control vs. PASS	1.3040	0.192	0.576	Ns	−0.097	Negligible
	Control vs. ECE	2.6095	0.009	0.027	*	−0.277	Small
	PASS vs. ECE	1.9074	0.056	0.169	Ns	−0.153	Small

Notes: PASS: Physical Activity and Sport Sciences degree; ECE: Early Childhood Education degree; Sd: Standard Deviation; Mg: Magnitude; *p*-values adjusted using Bonferroni correction. Significance levels are indicated as follows: * = *p* < 0.05; ** = *p* < 0.01. The interpretation of Cliff’s Delta follows established thresholds: negligible effect (<0.14), small effect (0.14–0.33), moderate effect (0.33–0.47), and large effect (>0.47).

**Table 5:** Intra-group comparison on each factor and total score of the disability attitudes questionnaire.

Factor 1: “Social and Interpersonal Relationships with People with Disabilities”							
	Time Moment	Sd	*p*-value	P_adj	Sig	Cliff’s Delta	Mg
Control	Pre-test vs. Post-test 2	−0.0543	0.9566	0.956	Ns	0.133	Negligible
PASS	Pre-test vs. Post-test 1	0.6740	0.5002	1.000	Ns	0.077	Negligible
	Pre-test vs. Post-test 2	0.6260	0.5312	1.000	Ns	0.133	Negligible
	Post-test1 vs. Post-test 2	−0.0832	0.9336	1.000	Ns	−0.058	Negligible
ECE	Pre-test vs. Post-test 1	0.4836	0.6286	1.000	Ns	0.077	Negligible
	Pre-test vs. Post-test 2	2.3326	0.0196	0.058	Ns	0.133	Negligible
	Post-test1 vs. Post-test 2	1.7880	0.0737	0.221	Ns	−0.058	Negligible
Factor 2: “Normalized Life”							
	Time Moment	Sd	*p*-value	P_adj	Sig	Cliff’s Delta	Mg
Control	Pre-test vs. Post-test 2	0.5475	0.584	0.584	Ns	0.325	Small
PASS	Pre-test vs. Post-test 1	3.7518	0.00017	0.0005	***	0.368	Medium
	Pre-test vs. Post-test 2	3.5157	0.00043	0.001	**	0.325	Small
	Post-test1 vs. Post-test 2	−0.4336	0.664	1.000	Ns	0.045	Negligible
ECE	Pre-test vs. Post-test 1	2.3729	0.0176	0.052	Ns	0.368	Medium
	Pre-test vs. Post-test 2	3.2124	0.0013	0.004	**	0.325	Small
	Post-test1 vs. Post-test 2	0.7555	0.449	1.000	Ns	0.045	Negligible
Factor 3: “Intervention Programs”							
	Time Moment	Sd	*p*-value	P_adj	Sig	Cliff’s Delta	Mg
Control	Pre-test vs. Post-test 2	0.2197	0.826	0.826	Ns	0.088	Negligible
PASS	Pre-test vs. Post-test 1	0.4399	0.659	1.000	Ns	0.093	Negligible
	Pre-test vs. Post-test 2	0.8346	0.403	1.000	Ns	0.088	Negligible
	Post-test1 vs. Post-test 2	0.3477	0.727	1.000	Ns	−0.006	Negligible
ECE	Pre-test vs. Post-test 1	1.2495	0.211	0.634	Ns	0.093	Negligible
	Pre-test vs. Post-test 2	1.0125	0.311	0.934	Ns	0.088	Negligible
	Post-test1 vs. Post-test 2	−0.2634	0.792	1.000	Ns	−0.006	Negligible
Total Score							
	Time Moment	Sd	*p*-value	P_adj	Sig	Cliff’s Delta	Mg
Control	Pre-test vs. Post-test 2	0.3816	0.7027	0.703	Ns	0.241	Small
PASS	Pre-test vs. Post-test 1	2.1456	0.0319	0.096	Ns	0.225	Small
	Pre-test vs. Post-test 2	2.2293	0.0257	0.077	Ns	0.241	Small
	Post-test1 vs. Post-test 2	−0.0415	0.9668	1.000	Ns	−0.029	Negligible
ECE	Pre-test vs. Post-test 1	1.3524	0.1762	0.528	Ns	0.225	Small
	Pre-test vs. Post-test 2	2.7998	0.0051	0.015	*	0.242	Small
	Post-test1 vs. Post-test 2	1.3742	0.1693	0.508	Ns	−0.029	Negligible

Notes: PASS: Physical Activity and Sport Sciences degree; ECE: Early Childhood Education degree; Sd: Standard Deviation; Mg: Magnitude; *p*-values adjusted using Bonferroni correction. Significance levels are indicated as follows: * = *p* < 0.05; ** = *p* < 0.01; *** = *p* < 0.001. The interpretation of Cliff’s Delta follows established thresholds: negligible effect (<0.14), small effect (0.14–0.33), moderate effect (0.33–0.47), and large effect (>0.47).

## Data Availability

Raw data supporting the reported results are available from the corresponding author upon request.

## Abbreviations

The following abbreviations are used in this manuscript:

PASS	Physical Activity and Sport Sciences
ECE	Early Childhood Education
SR	Social and Interpersonal relationships with people with disabilities
NL	Normalized Life
IP	Intervention Programs

## Appendix A

### Appendix A.1

**Table A1:** Content and Resources for the theoretical interventions.

Title	Contents	Resources
**Disability Contextualization**	Attitudes questionnaire 1. What is disability? What is illness? Is there a relationship between disability and illness? Inclusive Education Group reflection and presentation of main ideas. Analysis and reflection on stereotypes in videos. ONCE Foundation Campaign 2011.	YouTube videos and own elaboration slides
**Attitudes towards disability**	What are attitudes? Stereotypes Positive and negative attitudes Families of children with disabilities How to work on attitudes The daily attitude towards disability.	YouTube videos and own elaboration slides
**Inclusive Language Terms**	Inclusive Language CIDDM Classification. CIFS Classification. Origin of the deficits. Concepts of deficit, disability, and handicap. Therapeutic adaptations and process. Concept of normality	YouTube videos, google Jamboard and own elaboration slides
**Accessibility and supports**	Physic, sensorial and cognitive accessibility Support for people with disabilities Universal accessibility: The right of vote for people with intellectual disabilities. Conclusion of the training.	YouTube videos and own elaboration slides

**Table A2:** Sessions and content for the practical interventions.

Title	PASS	ECE
Practical Situation—Visual Disability	Contact with visual disability. Understanding the autonomy of visually impaired people. Learning to focus on possibilities rather than limitations. Showing that disability does not define ability.	Parts of the motor skills session: Warm-up exercises and connection activities. Motor skill exercises for inclusion in early childhood education. Contact with visual disability. Understanding the autonomy of people with visual disabilities. Focusing on possibilities rather than just limitations.
Faculty Accessibility Practice	Understanding the accessibility requirements of a building. Assessing the accessibility of a specific place for people with disabilities. Identifying accessibility challenges faced by disabled individuals.	
Cooperation and Observation Circuit	Exposure to various sensory and physical disabilities. Understanding the autonomy of people with disabilities Observing different attitudes toward disability.	Learning to interpret the specific curriculum for the Early Childhood Education stage in Extremadura. Understanding how to determine the educational objectives of motor skills sessions. Developing individual and group initiative. Learning to respect speaking turns.
Guiding a Blind Person	Learning to guide a blind person both in everyday context and during sports activities, specifically during running.	

### Appendix A.2

**Table A3:** Educational and curricular plans related to disability of PASS and ECE.

Degree	Comp.	Ac.Yr	Credits/Subjects	Description
Physical Activity and Sports Sciences	CT8	1st Year	18 Credits/ 3 Subjects	CT8: Promote equal opportunities and universal accessibility for people with disabilities and special populations in the field of physical activity and sports.
		2nd Year	42 Credits/ 7 Subjects	
		3rd Year	18 Credits/ 3 Subjects	
		4th Year	12 Credits/ 2 Subjects	
		Optatives	54 Credits/ 9 Subjects	
Early Childhood Education	CG3 CT3.6 CT8 CT12 CE70 CE81 CE83	1st Year	36 Credits/ 6 Subjects	CG3: Design and regulate learning environments within diverse contexts that address the unique educational needs of students, promote gender equality, equity, and respect for human rights. CT3.6: Critically and logically reflect on the need to eliminate all forms of discrimination, whether direct or indirect, particularly those based on race, gender, sexual orientation, or disability. CT8: Develop and demonstrate an ethical commitment in professional practice, a commitment that should foster the concept of holistic education with critical and responsible attitudes, ensuring effective equality between women and men, equal opportunities, universal accessibility for individuals with disabilities, and upholding the values of a culture of peace and democratic principles. CT12: Recognize the right to equal opportunities for individuals with disabilities and implement measures aimed at preventing or compensating for the disadvantages that may hinder their full participation in political, economic, cultural, and social life. CE.70: Understand the needs of children, accompanied by indicators of risk situations, to promote actions aimed at reducing the impact of disability on the child’s overall development. CE.81: Offer individualized educational measures through ICT to address diversity. CE83: Select conceptual and methodological tools to identify and address educational and organizational challenges in Early Childhood Education, developing flexible curricular projects capable of accommodating diversity.
		2nd Year	6 Credits/ 1 Subjects	
		3rd Year	24 Credits/ 4 Subjects	
		4th Year	N/A	
		Optatives	36 Credits/ 6 Subjects	

Comp.: Competences; Ac.Yr: Academic Year; CT: Transversal Competence; CG: General Competence; CE: Specific Competence.
